# Alert, but not alarmed - a comment on “Towards more accurate HIV testing in sub-Saharan Africa: a multi-site evaluation of HIV RDTs and risk factors for false positives (Kosack et al. 2017)”

**DOI:** 10.7448/IAS.20.1.22042

**Published:** 2017-06-19

**Authors:** Cheryl C. Johnson, Anita Sands, Willy Urassa, Rachel Baggaley

**Affiliations:** ^a^ Department of HIV, World Health Organization, Geneva, Switzerland; ^b^ Department of Essential Medicines and Health Products, World Health Organization, Geneva, Switzerland

**Keywords:** HIV, diagnostic, test, quality, misdiagnosis, false positive, Africa

Dear Editors,

We read with interest Kosack and colleagues’ article [1], which evaluated eight HIV rapid diagnostic tests (RDTs) using specimens collected from Médecins Sans Frontières (MSF) sites, between 2011 and 2015, in five African countries. Authors state that RDT accuracy differed from previous evaluations conducted by the World Health Organization (WHO), concluding only one HIV RDT achieved WHO prequalification performance criteria and none met WHO performance thresholds when using the “lower end of the 95% CI”. Authors attributed such “poor performance”, primarily poor specificity, to the RDTs and possible non-specific geographical and population-level interferrents.

Overall, we agree with the author’s findings and their conclusions, which affirm WHO recommendations to use a validated testing algorithm. However, we do not agree with the author’s discussion, which is at odds with WHO’s data and suggests misdiagnosis of HIV occurred and can be attributed to WHO-prequalified RDTs. Given the limitations of the evidence presented, we find their discussion to be potentially misleading.

The authors report that they found a substantial number of false reactive test results resulting in poor positive predictive values. This finding is based on the result of a single HIV RDT. A single reactive test result is never sufficient to make an HIV-positive diagnosis. To provide a definitive HIV diagnosis, WHO recommends countries use a high (≥5%) or low (<5%) prevalence testing strategy comprised of up to three RDTs as part of a validated testing algorithm [2]. Thus, it is incorrect for the authors to discuss these findings in the context of “misdiagnosis”, as no evidence of actual misdiagnosis (false positive or false negative diagnoses) is presented. In fact, while not noted by the authors, if all study sites used the data from Table 2 and adhered to WHO recommendations, all settings could construct a highly accurate testing algorithm and thereby provide highly accurate HIV diagnoses.

While the authors acknowledge that some studies have found HIV RDT performance, particularly specificity, to vary across populations and settings due to cross-reacting antibodies [3–9], they state that their study relied on self-reported morbidities and did not find a significant association with false reactive results. However, Table 7 suggests that self-reported “malaria” was associated with false reactive test results on one RDT (2.62, 95% confidence interval [CI]: 1.21–5.6). In the absence of a review of clinical records and patient information, it is not known what other morbidities or factors may have contributed to these results. Authors also did not provide details on the sites where the specimens were collected, or the testing algorithms used at each site. Without knowing the testing algorithms, it is not known if the specimens were characterized correctly.

Between 2011 and 2015 it is known that some of the study countries utilized a “tiebreaker” approach to resolve discrepant test results and rule in HIV infection, instead of considering these results as “inconclusive” and retesting patients in 14 days as recommended by WHO [2]. Previous studies have demonstrated this to be a possible cause of false positive results and misdiagnoses [10–18]. Thus, if these suboptimal testing strategies were used at site level, the study may have included “inconclusive” specimens that were misclassified as “HIV-positive” specimens. This could explain the high proportion of false reactive results observed.

Lastly, although the authors report this as a systematic head-to-head evaluation, it should be clarified that this study did not conduct a systematic head-to-head comparison of MSF and WHO evaluations. Authors do not discuss methodological differences between WHO performance evaluations and those presented by the authors; namely that WHO evaluations are conducted using specimens collected worldwide. WHO performance criteria are based on RDTs achieving ≥99% sensitivity and ≥98% specificity as a fixed proportion because characterized HIV-positive and HIV-negative specimens are used [19]. Using a 95% CI is only relevant if one is making an inference to the population where the specimens originated and the true HIV status of the specimens is unknown. Therefore, using the lower end of the 95% CI as a point of comparison between WHO and MSF evaluations is irrelevant and misleading.

This study presents important results and affirms the importance of using a validated testing algorithm, as recommended by WHO. While we concur with their conclusion, the author’s discussion points are not supported by the evidence in this article. Authors should reconsider their remarks or provide evidence indicating actual misdiagnosis, attributable to RDT performance alone.

This evidence should be viewed in the broader context of HIV diagnosis. Countries must be advised that WHO-prequalified HIV RDTs are accurate and can be used to provide a reliable HIV diagnosis. Nevertheless, countries and programmes need to ensure that they are following WHO-recommended HIV testing strategies and use a validated testing algorithm.
